# Spleen tyrosine kinase inhibition mitigates radiation-induced lung injury through anti-inflammatory effects and downregulation of p38 MAPK and p53

**DOI:** 10.3389/fonc.2024.1406759

**Published:** 2024-11-07

**Authors:** Guoxing Zhang, Ni Sun, Xiaohua Li

**Affiliations:** ^1^ Department of Intensive Care Unit, Jilin Province Tumor Hospital, Changchun, China; ^2^ Department of Infectious Diseases, The First Hospital of Jilin University, Changchun, China

**Keywords:** bioinformatics, radiation pneumonitis, Syk, p38 MAPK, p53

## Abstract

**Background:**

To explore new modulatory intervention targets for radiation-induced lung injury, bioinformatics analysis technology was used to search for the core driving genes in the pathogenesis of radiation pneumonitis, and the results were verified by a radiation-induced murine lung injury model to find possible new targets for the treatment of radiation lung injury.

**Method:**

Gene Expression Omnibus Database was used to identify differentially expressed genes in radiation pneumonitis. DAVID database was used for gene ontology (GO) and Kyoto Encyclopedia of Genes and Genome (KEGG) enrichment analysis. Gene Set Enrichment Analysis was used to analyze abnormal expressions. Protein–protein interaction networks were constructed using STRING and Cytoscape. Discovery Studio 4.5 software was used to find the preferred inhibitor of the specific gene. A radiation-induced lung injury model was induced in female C57BL/6N mice. The specific inhibitors were administered by intraperitoneal injection 24 h before and for 7 consecutive days after radiation. Lungs were harvested for further analysis 14 days and 10 weeks post-irradiation.

**Results:**

We screened Syk as one of the most important driver genes of radiation pneumonitis by bioinformatics analysis and screened the preferred Syk inhibitor fostamatinib from the drug database. Syk was highly expressed in irradiated lung tissue, and fostamatinib inhibited the level of Syk expression. Syk inhibitor significantly alleviated the radiation-induced lung injury and downregulated the increased expression of p38 MAPK, p53, IL-1β, and IL-6 in lung tissue at 2 weeks after radiation. The levels of TGF-β, COL1A1, and α-SMA and degree of pulmonary fibrosis at 10 weeks after radiation were also decreased by Syk inhibitor.

**Conclusion:**

Syk inhibitor may have a potential to be used as a targeted drug to mitigate radiation pneumonitis and inhibit radiation-induced pulmonary fibrosis.

## Introduction

Radiotherapy is an important treatment method for intrathoracic malignant tumor. Patients receiving radiotherapy on the chest are at a risk for radiation-induced lung injury (RILI) (1, 2). The lungs are moderately sensitive to radiation, and the lung tissue adjacent to the tumor may be damaged by radiation therapy. Alveolar type II cells are one of the most radiation-sensitive cells in lung tissue and are the earliest cells to undergo morphological changes after lung radiation. Radiation-associated pulmonary fibrosis may develop within 6 months, with increased interstitial myofibroblasts and collagen, intravascular fibrosis, and then a decrease in lung volume, and the patient then develops irreversible respiratory failure (3).

The development of radiation pneumonitis and pulmonary fibrosis seriously affects the quality-of-life of patients, and its poor prognosis imposes a burden on families and society. To date, there are no effective treatments for radiation pneumonitis and pulmonary fibrosis. Glucocorticoids can only alleviate the disease in the acute phase of radiation pneumonitis but cannot prevent the progression of pulmonary fibrosis (4). Therefore, it is of great clinical significance and value to study the pathogenesis of radiation pneumonitis, find effective drugs to treat radiation pneumonitis, and reduce the risk of radiation pulmonary fibrosis.

Bioinformatic methods, especially in combination with microarray technology, provide new avenues to study the molecular pathogenesis of various diseases through data mining at the molecular level (5, 6).

Spleen tyrosine kinase (Syk) is a cytoplasmic tyrosine kinase which contains two SRC homology 2 (SH2) domains and a kinase domain (BOX 1), and it plays an essential role in signal transduction initiated by the classic immunoreceptors, including B-cell receptors (BCRs), Fc receptors, and the activated natural killer receptors (7). Syk is highly expressed in hematopoietic cells (8), including B cells, immature T cells, mast cells, neutrophils, platelets, macrophages, monocytes, and non-immune osteoclasts (9). As most of these cells are involved in the instigation and establishment of tissue pathology in autoimmune allergic and autoinflammatory diseases, patients who are suffering from RA, SLE, ITP, AHA, and allergic rhinitis stand a good chance to benefit from Syk inhibition (7). Several recent studies have found that Syk had other functions beyond immune regulation. Syk has been shown to promote pulmonary angiogenesis by activating downstream ERK1/2 and Akt signaling pathways in a rat hepatopulmonary syndrome (HPS) model (10) and to promote platelet-derived-growth factor-BB-induced proliferation of rat pulmonary vascular smooth muscle cells (11, 12). Syk inhibition could prevent toxin-induced liver fibrosis, associated hepatocellular damage and intra-hepatic inflammation, and hepatocarcinogenesis (12, 13). However, the involvement of Syk in the regulation of p38 and p53 in radiation pneumonitis and radiation pulmonary fibrosis has not been investigated. In the present study, we evaluated the effects of Syk inhibitor on radiation-related lung injury in a mouse model.

## Materials and methods

### Bioinformatics analysis

The mRNA micro-array datasets used in biological information analysis are GSE85359 and GSE41789. These data were based on the Agilent GPL1261 platform ([mouse430_2] Affymetrix mouse genome 430 2.0 array), and a total of 50 radiation pneumonitis samples and 22 normal control samples were included. Differentially expressed genes (DEGs) in radiation pneumonitis were identified using Gene Expression Omnibus (GEO) database. DAVID database was used for gene ontology (GO) and Kyoto Encyclopedia of Genes and Genome (KEGG) enrichment analysis, and then Gene Set Enrichment Analysis (GSEA) was used to analyze abnormal expressions. Protein–protein interaction (PPI) networks were constructed using STRING (String: http://string.embl.de/) and Cytoscape. Syk inhibitors were analyzed using Discovery Studio 4.5 software.

### Animals

C57BL/6N mice (female, 6–8 weeks) purchased from Charles River Laboratories (China, Beijing) were raised in specific pathogen-free (SPF) barrier environment in the Laboratory Animal Center of Jilin University with suitable temperature, humidity, and light control. Animal experiments were carried out in accordance with the ethical approval issued by the Animal Ethics Committee at the First Bethune Hospital of Jilin University (IACUC approval number 2022-0034).

### Antibodies and reagents

Syk inhibitor fostamatinib was purchased from Invitrogen™ (Carlsbad, CA, USA), interleukin-1β (IL-1β), interleukin-6 (IL-6), phospho-p38 (p-p38), p53, α-smooth muscle actin (α-SMA), collagen type 1-alpha1 (COL1A1), transforming growth factor (TGF)-β, and Syk antibodies were purchased from ZEN BioScience (Chengdu, Sichuan, China), and the p-Syk antibody was from the β-tubulin antibody purchased from Abcam (Cambridge, MA, USA). Hydroxyproline content assay kit was purchased from Solarbio (cat# BC0255). All other reagents were obtained from Sigma Chemical Co. (St. Louis, MO, USA).

### Mouse model of radiation pneumonitis

The female C57BL/6N mice were anesthetized by an intraperitoneal injection of pentobarbital (60 mg/kg). A total of 30 were fixed and placed in the supine position for radiation. The mouse head and the abdomen were shielded by using lead plates. Thoracic radiation was performed with a single dose of 18 Gy in an X-ray irradiator (Faxitron, USA). The mice were randomly allocated into group R (*n* = 10) and group R+S (intraperitoneal injection of 20 mg/kg·day fostamatinib, *n* = 10; fostamatinib was dissolved in 0.5% DMSO). The other 10 untreated mice served as controls (group C). The mice in groups C and R received an equal volume of 0.5% DMSO simultaneously. All mice received intraperitoneal injection 1 day before radiation and after 7 days. Five mice in each group were randomly selected and sacrificed 2 weeks after radiation. The remaining mice were sacrificed at 10 weeks after radiation by cervical dislocation.

### Lung tissue histology and immunohistochemistry

The mice were sacrificed, and both lungs were harvested. Part of the lungs were snap-frozen in liquid nitrogen for western blot analysis. Some lungs were fixed in 10% neutral-buffered formalin at 4°C for 12 h, processed, embedded in paraffin wax, sliced into 5-μm sections, and observed by using a light microscope after hematoxylin/eosin (HE) and Masson’s trichrome staining. The number of inflammatory cells per high-power field, width of blood vessels, and width of interalveolar septa were quantitatively determined from five random fields in high magnification. Smith scale was applied to evaluate the degree of lung injury at 2 weeks after radiation, according to the presence of pneumonedema, inflammation, hemorrhage, atelectasis, and hyaline membrane formation. Each of these features was scored as follows: no damage (0), mild (1), moderate (2), and severe (3) (14, 15). Each mouse was observed at five high-power fields, and then the average was taken. Ashcroft score was used to assess the grade of lung fibrosis at 10 weeks after radiation (16, 17).

The immunohistochemistry of Syk was performed as described previously (18). The slides were treated overnight with primary antibody SYK (1:50) at 4°C and incubated with secondary antibody (1:500) (HRP-conjugated anti-rabbit IgG).

### Western blot analysis

Total cellular protein was extracted as previously described. Equal amounts of proteins were mixed with an equal volume of reducing SDS sample buffer and boiled at 95°C for 5 min. Protein samples were resolved by 10% SDS-PAGE and electroblotted on nitrocellulose membranes (BioRad, Hercules, CA, USA). After electroblotting, nonspecific binding was blocked with 5% bovine serum albumin (BSA). The membrane was incubated overnight with primary antibodies at 4°C and incubated with horseradish-peroxidase-conjugated secondary antibodies for 1 h at room temperature. Primary antibodies against the following proteins were used at 1:1,000 dilutions unless otherwise indicated: p-SYK, IL-1β, IL-6, TGF-β1 p-p38, p53, α-SMA, COL1A1, and GAPDH antibodies (1:5,000 or 1:10,000). During the western blot experiments, some blots were cut prior to hybridization with antibodies. Immunoreactive bands were visualized using enhanced chemiluminescence (Amersham, Arlington, IL, USA) as previously described. Western blot was repeated at least five times.

### Statistical analysis

All experiments were conducted at least three times. Data depicted in graphs represent the mean ± SE for each group. Brown–Forsythe and Bartlett tests were employed to assess the homogeneity of variances. One-way analysis of variance (ANOVA) was conducted for comparison among multiple groups, and Tukey multiple comparison test was used to evaluate the significance between groups. In all analyses, *P*-values less than 0.05 were considered statistically significant.

## Results

### Syk was identified as one of the most important core driving genes of radiation pneumonitis by bioinformatics analysis

**Figure 1:**
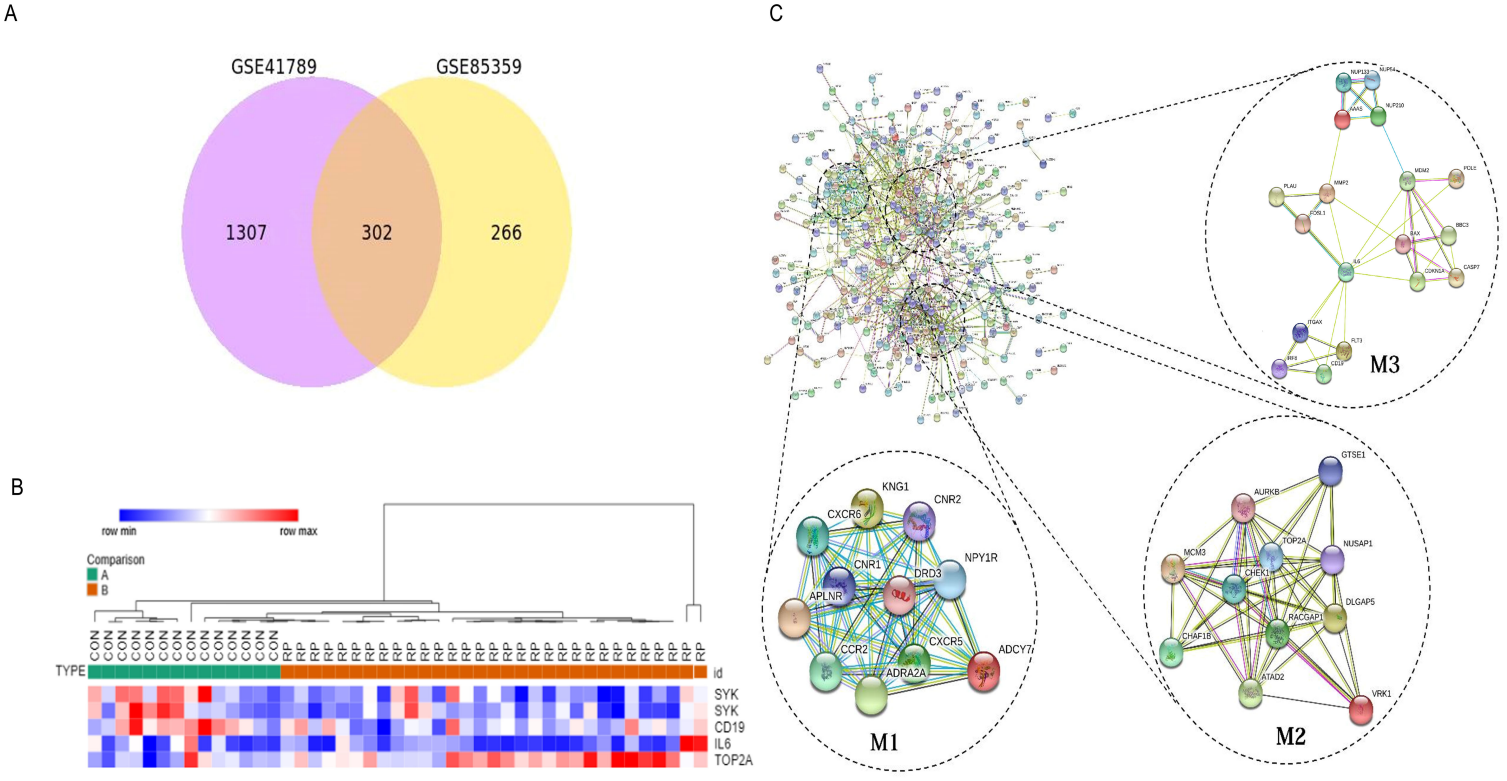
Identify the core driving genes of radioactive pneumonitis from differentially expressed genes (DEGs). **(A)** Venn diagram of differentially expressed genes in the three groups. **(B)** Heatmap of the core genes of radioactive pneumonitis. **(C)** Protein network interaction diagram of DEGs, mainly three modules.

The Venn diagram in **Figure 1A** shows the DEGs between radioactive pneumonitis and normal lung tissue, and the heatmap of core genes is shown in **Figure 1B**. We constructed a PPI network and then performed molecular complex detection (MCODE) to screen out PPI network modules with MCODE score >10 and number of nodes >11. The core driver genes of radiation pneumonitis were screened and are listed in **Table 1**. Among these genes, Syk was highly expressed, with a node degree of 48 in radiation pneumonitis. By analyzing the PPI network of all DEGs and using MCODE to screen out the three most important modules (as shown in **Figure 1C**), we annotated the functions of these module genes and their abundances (as shown in **Supplementary Table S1**), including leukocyte chemotaxis, cell cycle, integral component of membrane, mitotic cytokinesis, ATP binding, and P53 signaling pathway.

**Table 1:** Key nodes in the protein–protein interaction network with degrees >15 of radiation pneumonitis.

Name	Degree	Betweenness
SYK	48	0.269269
CD19	46	0.187161
IL6	31	0.195447
TOP2A	27	0.035771
AURKB	26	0.063969
ITGAX	25	0.029731
KNG1	23	0.031814
MDM2	22	0.076925
CD79A	20	0.029324
CHEK1	20	0.040371
CXCR5	19	0.017667
MCM3	18	0.027609
IL7R	18	0.007422
FASLG	17	0.030839
ADCY7	17	0.03224
HDAC9	17	0.065686
TAC1	16	0.016625
MAPK11	16	0.057158
CNR1	16	0.013967
CXCR6	16	0.00766
BAX	16	0.036423
PLEK	16	0.068431
DNAH8	16	0.07225
CDKN1A	15	0.031859
CCR2	15	0.008314

The GO analysis showed that the DEGs between radioactive pneumonitis and normal lung tissue were enriched in B-cell receptor signaling pathway, transmembrane receptor protein tyrosine kinase signaling pathway, integrin-mediated signaling pathway, positive regulation of cytokinesis, and mobile behavior. The KEGG analysis revealed enrichment of DEGs in chemokine signaling pathway, cytokine–cytokine receptor interaction, P53 signaling pathway, primary immune deficiency, and hematogenesis cell lines (**Supplementary Figure S1**, **Supplementary Table S2**). The GSEA of DEGs showed that there was a significant abnormal regulation of mechanical ventilation pathways in radiation pneumonitis (**Supplementary Figure S2**).

### Fostamatinib was screened as an ideal drug targeting Syk inhibition

**Figure 2:**
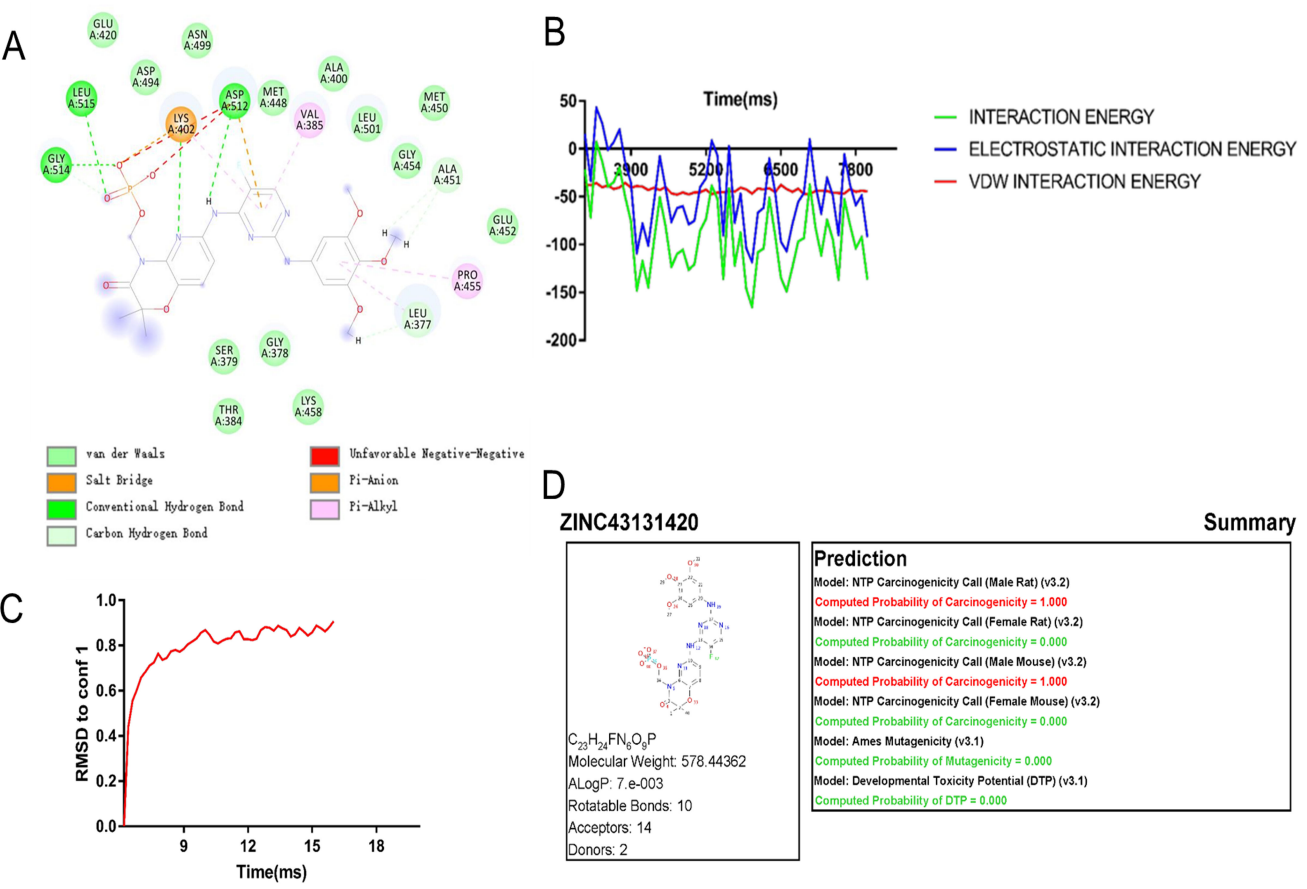
Drug molecule action mechanism analysis and toxicity analysis of fostamatinib. **(A)** Fostamatinib binds to SYK transcription protein through hydrogen bond and van der Waals force. **(B)** Interaction energy curves between drug molecule and SYK transcription protein. **(C)** RMSD curve of ZINC43131420 (fostamatinib). **(D)** Toxicology report of fostamatinib.

We conducted drug molecular docking analysis targeting the inhibition of Syk gene in the drug database. The criteria of drug screening include the following: (1) it has the ability to specifically kill the cells with abnormal expression of Syk and has already entered in clinical trials, (2) minor toxicity and side effects to the human body, and (3) good solubility and high absorption. The top 20 drug molecules scored out of 7,049 drugs that specifically act on the SYK transcriptional protein were selected and are presented in **Supplementary Table S3**, among which ZINC43131420 (fostamatinib) had the most favorable pharmacokinetic profile and less hepatotoxicity according to ADMET analysis (**Table 2**). Fostamatinib binds to SYK transcriptional protein primarily through hydrogen bonding and van der Waals forces (**Figure 2A**). The intermolecular potential energy curves revealed the potential energy changes between the drug and target protein molecules (**Supplementary Figure S3**). The interaction energy curve and RMSD curve (**Figures 2B, C**) indicated that fostamatinib is very stable and not prone to be allosteric. The toxicity analysis results are shown in **Figure 2D**. Fostamatinib has no potential of mutagenic and developmental toxicity, which could be an ideal drug.

**Table 2:** ADMET analysis of the top 20 medicines from drug screening docking analysis.

Name	Visible	ADMET_ BBB_Level	ADMET_ Absorption_Level	ADMET_ Solubility_Level	ADMET_ Hepatotoxicity
ZINC000095099885	False	4	3	0	1
ZINC000008215434	False	4	3	0	1
ZINC000008216447	False	4	3	1	1
ZINC000096014967	False	4	3	0	1
ZINC43131420	True	4	2	2	1
ZINC000028538573	False	4	2	0	1
ZINC000196105913	False	4	3	0	1
ZINC000072131515	False	4	3	0	0
ZINC000014883350	False	4	3	0	0
ZINC000008219152	False	4	3	2	0
ZINC000008216371	False	4	3	0	1
ZINC000008551971	False	4	3	0	1
ZINC000008215403	False	4	3	2	1
ZINC000001540640	False	4	0	2	1
ZINC000008217042	False	4	3	1	1
ZINC000013130935	False	4	2	1	1
ZINC000002526388	False	4	0	2	1
ZINC000100774817	False	4	2	1	1
ZINC000014883348	False	4	3	0	0
ZINC000030729923	False	4	3	0	0

### Syk inhibitor attenuated lung pathology

**Figure 3:**
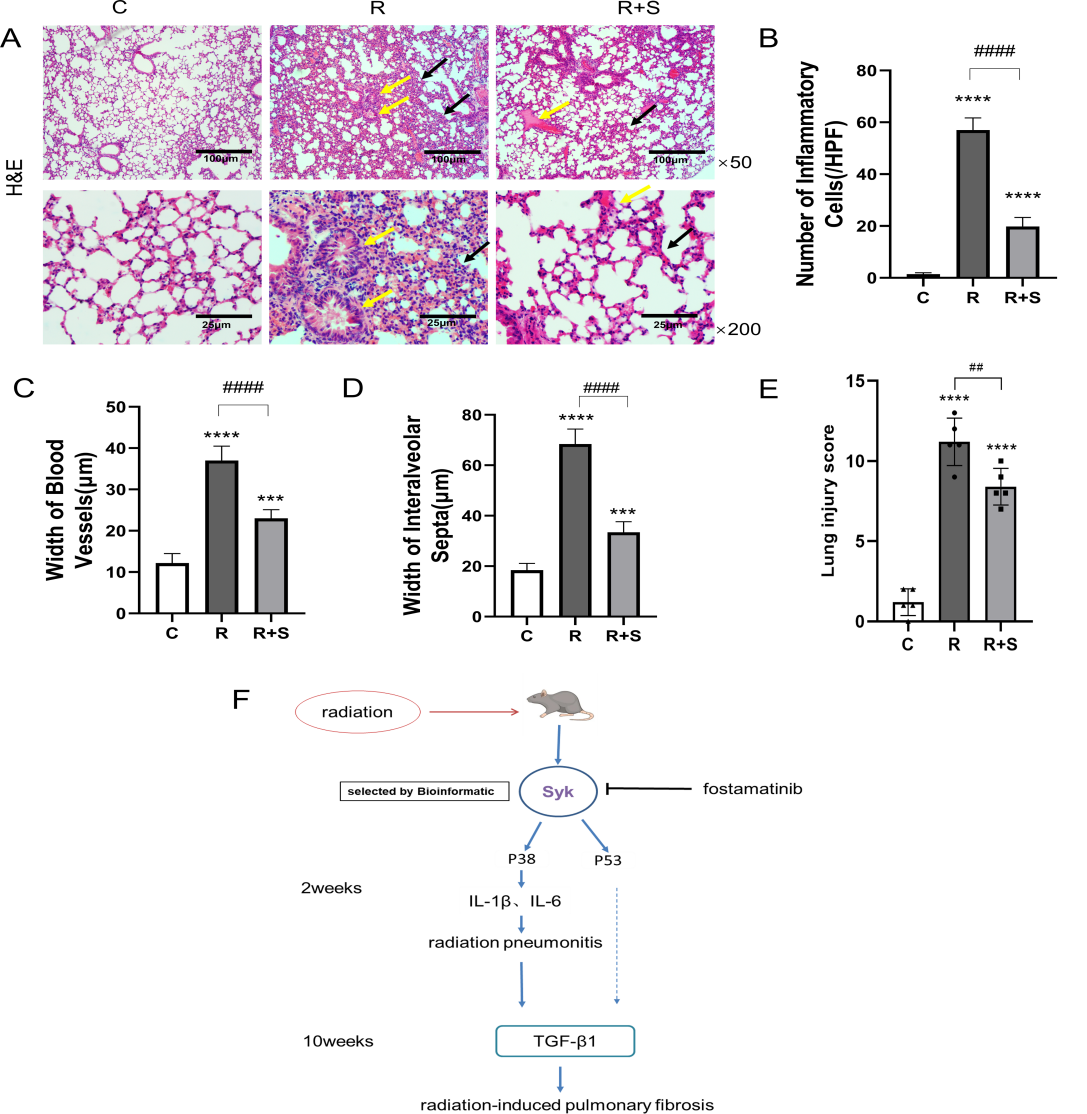
Pathological changes of the lung at 2 weeks after radiation. **(A)** Photomicrographs show the H&E staining of lung tissues of mice in different groups at ×50 and ×200 magnification. Scale bar, 100 μm for ×50 and 25 μm for ×200. **(B–D)** The number of inflammatory cells per high-power field of vision, width of blood vessels, and width of interalveolar septa were determined from five random fields in high magnification in lung tissue (*n* = 5 for each group). **(E)** H&E staining area from five random fields in high magnification was analyzed, and Smith scale was used to evaluate the degree of lung injury (*n* = 5 for each group). **(F)** Diagram of the hypothetical mechanism by which Syk and its inhibition regulate radiation-related lung injury. ^***^ *P* < 0.0005, ^****^ *P* < 0.00005 vs. C group, ^##^ *P* < 0.005, ^####^ *P* < 0.00005. HPF, high-power field.

We observed the pathological changes in the lung tissue of mice at 2 weeks after radiation. Compared with group C, the alveolar walls were significantly widened, the capillaries were dilated and congested (yellow arrows), and there were inflammatory cell infiltration around bronchioles and pulmonary interstitial inflammation (black arrows) in group R (**Figures 3A–D**). The application of Syk inhibitor significantly reduced pulmonary interstitial inflammation, slightly widened alveolar walls, and reduced telangiectasia and inflammatory cell exudation (**Figures 3A–D**). Lung injury score confirmed that there was a statistical decrease in group R+S compared with group R (**Figure 3E**). Therefore, it can be inferred that Syk inhibitors have a protective effect on radiation pneumonitis.

### Syk inhibitor decreased the expression of p-p38 and p53 in the lung tissue of radiation pneumonitis

**Figure 4:**
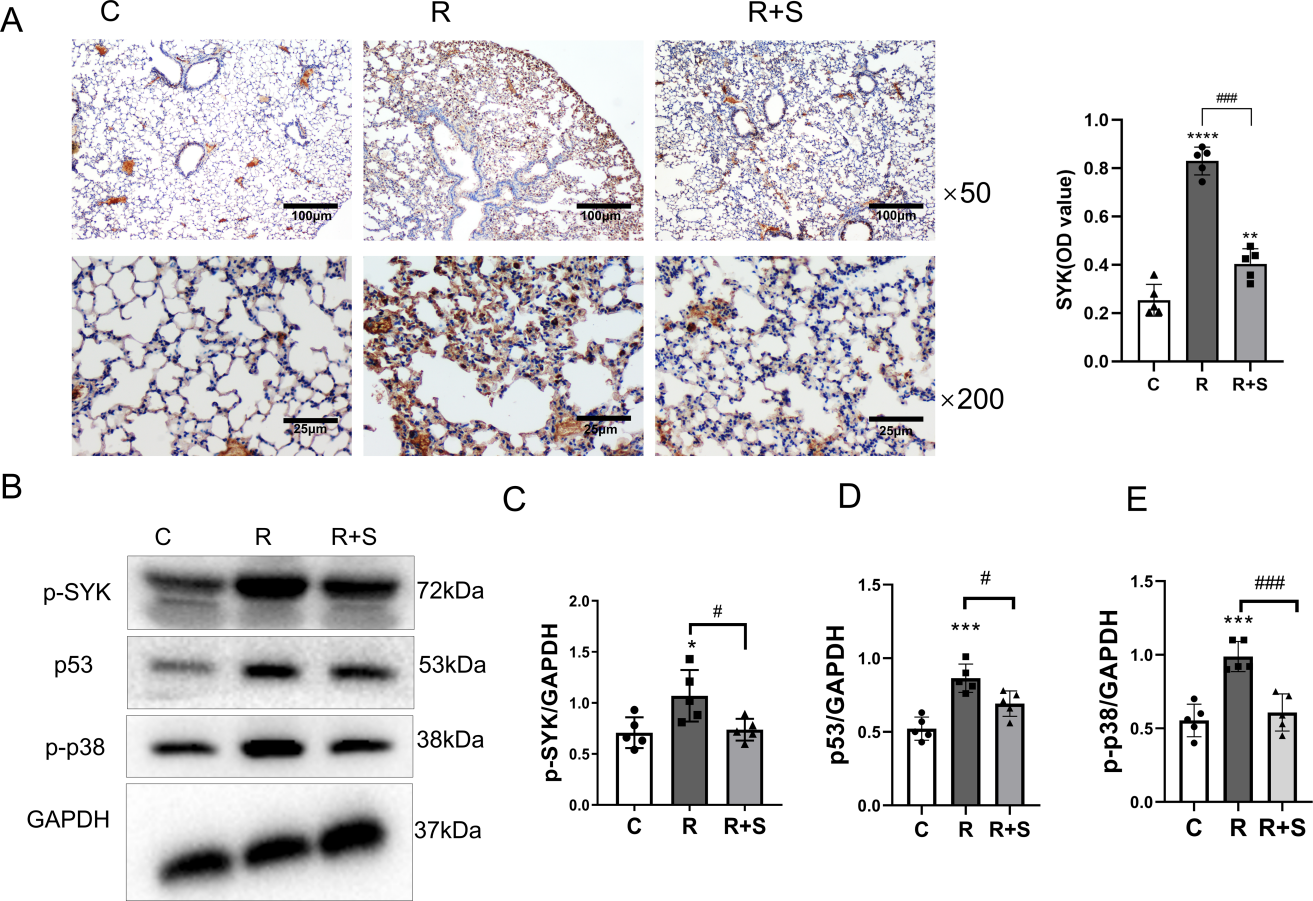
Relative expression of Syk, p-p38, and p53 in lung tissue at 2 weeks after radiation. **(A)** Immunohistochemical staining for Syk in lung sections from different groups at ×50 and ×200 magnification. Scale bar, 100 μm for ×50 and 25 μm for ×200. **(B)** The protein levels of p-Syk, p53, and p-p38 in lung tissues from the indicated mouse groups were detected by using Western blot. Typical images are shown. **(C–E)** Relative protein expression levels of p-Syk, p53, and p-p38 in lung tissue from different parts of the same gel were compared among different groups. ^*^ *P* < 0.05, ^***^ *P* < 0.0005, ^****^ *P* < 0.00005 vs. C group; ^#^ *P* < 0.05; ^###^ *P* < 0.0005.

Consistent with the results of the bioinformatics analysis, we found that the protein level and phosphorylation of Syk were highly expressed in the lung tissue of irradiated mice and significantly decreased in mice administered with the Syk inhibitor (**Figures 4A–C**).

Western blot analysis demonstrated that the expression of p-p38 and p53 in lung tissue increased at 2 weeks after radiation compared with that observed in the control group, which was also consistent with the findings in the bioinformatics analysis. p-p38 and p53 were downstream sites of the Syk signaling pathway, and their expressions decreased in the lung tissue of the group administered with the Syk inhibitor (**Figures 4D, E**).

### Syk inhibitor suppressed the expression of IL-6 and IL-1β in the lung tissue of radiation pneumonitis

**Figure 5:**
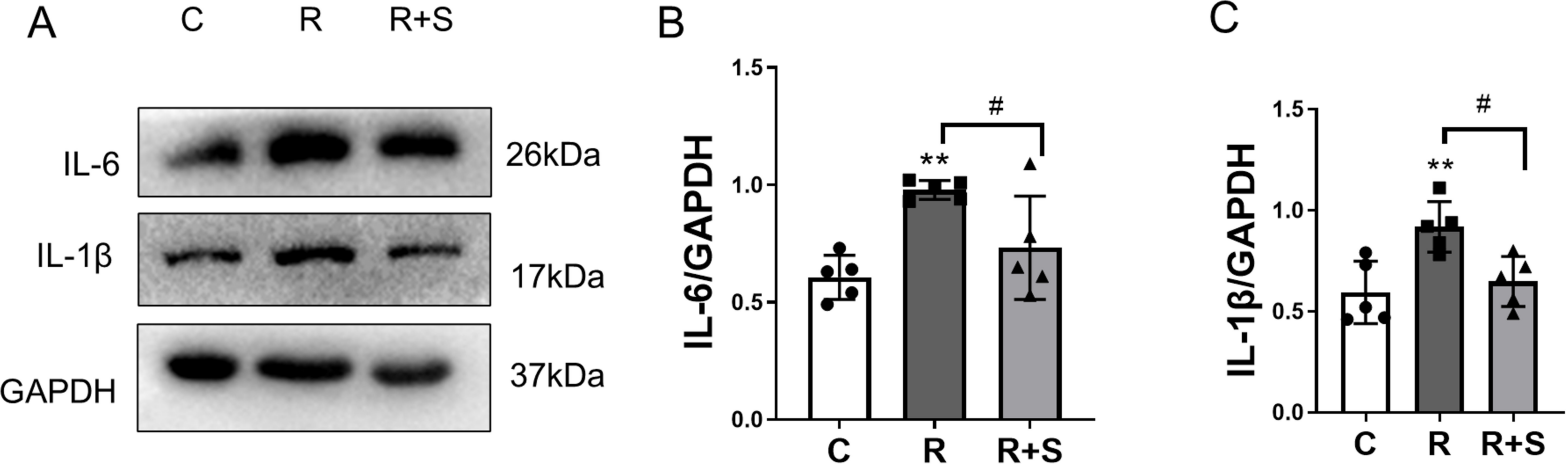
Relative expression of IL-1β and IL-6 in lung tissue at 2 weeks after radiation. **(A)** The protein levels of IL-6 and IL-1β in lung tissues from the indicated mouse groups were detected by using Western blot. Typical images are shown. **(B, C)** The relative protein expression levels of IL-1β and IL-6 from different parts of the same gel in lung tissue were compared among the different groups. Each bar represents the mean ± S.E. **P < 0.005 vs. C group; ^#^ *P* < 0.05.

IL-1β and IL-6 are the major cytokines in radiation pneumonitis (19, 20). To determine whether inflammation was suppressed by Syk inhibitor, the expression of IL-1β and IL-6 at 2 weeks after radiation was evaluated by Western blot analysis. The levels of IL-6 and IL-1β expressions in lung tissue were significantly increased in irradiated lung tissue and were downregulated in the Syk-inhibitor-administered group, as shown in **Figures 5A–C**.

### Syk inhibitor attenuated radiation-induced pulmonary fibrosis

**Figure 6:**
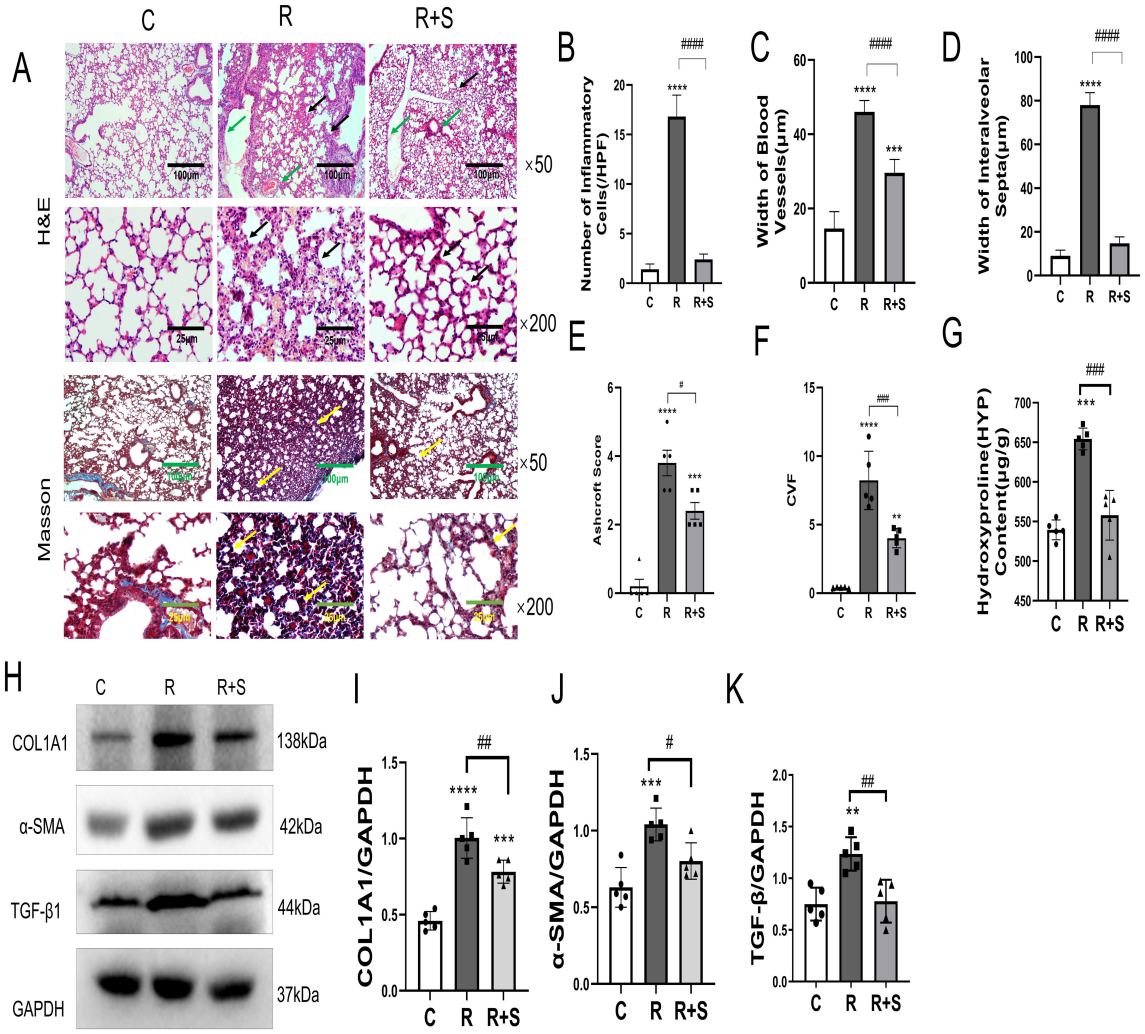
Pathological changes and relative expression of hydroxyproline content, COL1A1, α-SMA, and TGF-β in the lung at 10 weeks after irradiation. **(A)** Photomicrographs at ×50 and ×200 magnification shows H&E staining and Masson’s trichrome staining of the lung tissue of mice in the different groups. Scale bar, 100 μm for ×50 and 25 μm for ×200. **(B–D)** The number of inflammatory cells per high-power field of vision, width of blood vessels, and width of interalveolar septa were determined from five random fields in high magnification in lung tissue. **(E)** Ashcroft score was used to evaluate the degree of lung fibrosis. **(F)** Collagen volume fraction (CVF) in Masson staining in different groups. **(G)** Hydroxyproline content in lung tissues in the different groups. **(H)** The protein levels of COL1A1, α-SMA, and TGF-β in lung tissues from the indicated mouse groups were detected by using Western blot. Typical images are shown. **(I–K)** The relative protein levels of COL1A1, α-SMA, and TGF-β in lung tissues were compared between different groups. ^**^ *P* < 0.005; ^***^ *P* < 0.0005, ^****^ *P* < 0.00005 vs. C group; ^#^ *P* < 0.05; ^##^ *P* < 0.005; ^###^ *P* < 0.0005. HPF, high-power field.

The experimental results discussed above indicated that Syk inhibitors can alleviate radiation pneumonitis. We further investigated whether pulmonary fibrosis was influenced by Syk inhibition. Mice were sacrificed at 10 weeks after radiation. H&E and Masson’s trichrome staining showed that, in group R, the alveolar walls were widened, the capillaries were moderately dilated and congested (green arrows), there was some lymphocyte and monocyte infiltration (**Figures 6A–D**), and mild to moderate fibrous and collagen hyperplasia was observed, with alveolar collapse and poor aeration (black arrows). Compared with group R, the degree of lung interstitial inflammation and fibrous and collagen hyperplasia (black arrows) was significantly reduced in the Syk-inhibitor-administered group (**Figures 6A–D**). The Ashcroft score and collagen volume fraction (CVF) confirmed changes in pulmonary fibrosis in these three groups (**Figures 6E, F**). The content of hydroxyproline in the lung tissue of group R was significantly higher (yellow arrows) than that of the control group, suggesting collagen hyperplasia, which was significantly reduced by Syk inhibitor (**Figure 6G**).

TGF-β is an important cytokine that promotes the pathogenesis of pulmonary fibrosis by inducing the production of collagen and the loss of lung elasticity (21). α-SMA and COL1A1 are usually used as biomarkers to evaluate the degree of pulmonary fibrosis (22, 23). We demonstrated that the expressions of TGF-β1, SMA, and COL1A1 in the lung tissue were significantly increased in group R and were inhibited by Syk inhibitor in group R+S (**Figures 6H–K**). The results showed that Syk inhibitor could attenuate the development of pulmonary fibrosis.

## Discussion

At present, it is believed that the occurrence of radiation pneumonitis is closely related to the damage of alveolar type II cells and vascular endothelial cells. Damage of alveolar type II cells affects alveolar tension, leading to lung compliance decrease, alveolar collapse, and atelectasis. The damage of vascular endothelial cells leads to increased vascular permeability and microthrombus formation. In previous studies, Syk has been shown to promote pathological pulmonary angiogenesis and the proliferation of pulmonary vascular smooth muscle cells (9–11). However, the role of Syk in radiation pneumonitis has not been characterized. By comparing radiation pneumonitis with normal lung tissue using bioinformation technology, spleen tyrosine kinase (Syk) was identified as one of the most important driving genes of radiation pneumonitis, and the safest and most effective Syk inhibitor, fostamatinib, was selected as the inhibitor. Rube, CE and colleagues reported that the expression of the pulmonary inflammatory cytokine TGF-β significantly increased 12 h after irradiation and then returned to normal within a week, which is the so-called latent period. The most significant increase in cytokine occurred 2–4 weeks after irradiation (24). Consequently, administering fostamatinib before and for 7 days after exposure may potentially reverse or inhibit the evolution of radiation pneumonitis. Our study preliminarily demonstrated the important role of Syk in the pathogenesis of radiation pneumonitis. Inhibition of Syk could significantly reduce the degree of radiation pneumonitis and pulmonary fibrosis, and its effect was mediated in part by downregulating the activation of p-p38 and p53 (**Figure 3F**).

The DEGs between radiation pneumonitis and normal lung tissues were obtained by bioinformatics analysis, and the core driver genes were screened, among which Syk had the highest interaction and node degree. Mathew, B. et al. reported Syk as one of the most important regulatory nodes in activated radiation-induced lung injury network through a topological analysis of gene product interaction networks (18), which is consistent with our result. Furthermore, the results of GO and KEGG enrichment analysis revealed that DEGs were mainly enriched in B cell receptor signaling pathway, B cell receptor complex, chemokine signaling pathway, primary immunodeficiency, cytokine–cytokine receptor interaction, etc. It indicated that the occurrence of radiation pneumonitis was directly related to the abnormal immune response. Syk is an immune receptor-associated protein tyrosine kinase with the highest expression in hematopoietic cells and is involved in the development and proliferation of B cells (8). It has a good specific activation effect on classical immune receptors such as B-cell receptors and active Fc receptors (FcRs) (7), and it is one of the important therapeutic targets for autoimmune diseases. B-cell receptor activation stimulates downstream cascade that involves Syk activation and, subsequently, B-cell activation (25). Syk is also expressed in many other types of cells, such as fibroblasts, epithelial cells, and vascular endothelial cells (26). Several studies have shown Syk as an important therapeutic target for inflammatory diseases (27, 28)—for instance, Syk was found to be activated at high levels in asthma patients and mouse models, and Syk inhibitor showed potent synergistic anti-inflammatory effects (29). In another study, the expression of total and phosphorylation levels of Syk was increased in patients with IgA nephropathy, and Syk inhibitor attenuated IgA-induced inflammation in tubular cells (30). In a recent study, the Syk inhibitor fostamatinib prevented skin and lung fibrosis and inflammation in bleomycin-induced systemic scleroderma mice, and the anti-fibrotic effects of fostamatinib were associated with decreased Syk phosphorylation and TGF-β expression (31). For the first time, we discovered and demonstrated Syk as a therapeutic target in radiation pneumonitis and pulmonary fibrosis.

The results of GO and KEGG enrichment analyses showed that DEGs were enriched in the p53 signaling pathway, ligase activity, and positive regulation of cytokinesis, indicating that radiation pneumonitis was associated with abnormal cell division. p53 is involved in cell cycle regulation and plays an important role in cell growth and inhibiting tumor proliferation. When cell DNA is damaged, the p53 gene exhibits the role of an oncogene to promote abnormal cell proliferation, resulting in a malignant phenotype of all cells (32). Furthermore, a recent study determined that senescence, rather than loss, of alveolar type II cells promoted progressive fibrosis and showed that interventions targeting p53 activation could block pulmonary fibrosis (33). The connection between SYK and p53 has been studied earlier. Inhibiting SYK *in vitro* downregulated the expression of p53 in pro-B-ALL (SEM cells) (34), HCT116, and HT1080 cell lines (25), which highlights the effect of SYK inhibition on p53 expression and activity. Our animal experiments showed that p53 expression was elevated in radiation pneumonitis, which was consistent with the bioinformatics results, whereas inhibition of Syk downregulated p53 expression.

p38, a member of the mitogen-activated kinase (MAPK) family, plays an essential role in inflammation and host defense in immunity by regulating the production of pro-inflammatory cytokines and chemokines, such as IL-6 and IL-1β (35, 36). In B cells stimulated by oxidative stress, Syk was required for p38 activation and the regulation of cell cycle progression (37). Syk inhibitor could improve glomerulosclerosis of lupus nephritis and inhibit the increase of TGF-β1 mRNA levels and p38MAPK signaling pathways in the kidney (38). Inhibition of Syk *in vitro* could attenuate the epidermal growth factor-induced activation of p38 MAPK in A431, CAL27, and SAS cells (39). In our experiment, the increase of p-p38 expression in radiation pneumonitis was downregulated in Syk-inhibitor-treated mice.

IL-1β, produced by a variety of pulmonary cells in response to a microbial challenge, drives the inflammation cascade, localizes neutrophils, and promotes the production of inflammatory modulators, such as IL-6 production (40, 41). IL-6 is a pleiotropic cytokine that is commonly produced at local tissue sites and released into circulation in almost all situations of homeostatic perturbation, typically including endotoxemia, endotoxic lung, trauma, and acute infections (42, 43). In our studies, Syk inhibitor treatment could downregulate the levels of pro-inflammatory cytokines IL-1β and IL-6 (44). Syk inhibitor may treat radiation pneumonitis by reducing the expression of pro-inflammatory factors through the p38 pathway.

Inflammatory cells and macrophages at the injury site after ionizing radiation induce the synthesis and release of cytokines, promote the differentiation of fibroblasts into myoblast fibroblasts, stimulate the formation of collagen and fibronectin, and accelerate the deposition of extracellular matrix, which inhibits matrix degradation, leading to the formation of pulmonary fibrosis (45–47). Myofibroblasts are the main cells that result in the abnormal deposition of extracellular matrix and play an important role in the formation of pulmonary fibrosis. TGF-β plays an essential role in the development of pulmonary fibrosis (48). The p38MAPK signaling pathway contributes to TGF-β-induced fibroblast differentiation (49). α-SMA mainly exists in the cytoplasm of myofibroblasts and is the main marker molecule of myofibroblasts. COL1A1 is the main component of collagen. In our study, α-SMA and COL1A1 were measured to assess the degree of pulmonary fibrosis. Our results showed that the elevated levels of TGF-β, α-SMA, and COL1A1 in irradiated lung tissue were significantly reduced with Syk inhibitor administration, and the degree of fibrosis was also reduced by histopathological examination.

## Conclusions

Syk inhibitor alleviated the pneumonitis and fibrosis caused by radiation through decreasing the expression of p38 MAPK and p53 and downregulation of pro-inflammatory cytokines such as IL-1β and IL-6 and pro-fibrotic cytokine TGF-β. Syk inhibitor may have the potential to be a targeted drug in the intervention of radiation-induced lung injury.

## Data Availability

The datasets presented in this study can be found in online repositories. The name of the repository and accession number can be found below: https://doi.org/10.6084/m9.figshare.20113898.v1.
