# Supplementary material for: Spleen tyrosine kinase inhibition mitigates radiation-induced lung injury through anti-inflammatory effects and downregulation of p38 MAPK and p53
Source: Front Oncol. 2024 Nov 7;14:1406759. doi: 10.3389/fonc.2024.1406759 (PMC11578954; doi:10.3389/fonc.2024.1406759)
Supplement: Supplementary file 1 [file DataSheet1.pdf]

Supplemental Table 1. Functional and pathway enrichment analysis of the module genes

Module	Term	Count	%	P value	FDR
Module 1	GO:0030595~leukocyte chemotaxis	2	11.40	5.75E-03	3.84
	GO:0016021~integral component of membrane	5	28.51	5.26E-02	25.60
	GO:0004949~cannabinoid receptor activity	2	11.40	3.28E-03	1.42
	GO:0000281~mitotic cytokinesis	2	10.13	7.30E-03	6.33
Module 2	GO:0005634~nucleus	7	35.46	2.23E-04	0.16
	GO:0005524~ATP binding	6	30.40	5.14E-04	0.33
	xtr04110: Cell cycle	3	15.20	2.00E-03	0.63
	GO:0051281~positive regulation of release of sequestered calcium ion into cytosol	2	6.89	2.14E-02	23.01
Module 3	GO:0005643~nuclear pore	2	6.89	2.32E-02	18.54
	GO:0004252~serine-type endopeptidase activity	2	6.89	9.30E-02	51.89
	ocu04115: p53 signaling pathway	5	17.24	1.90E-05	0.02

Supplemental Table 2. Functional and pathway enrichment analysis of hub genes of Radiation Pneumonitis

Category	Term	Count	%	P Value
GOterm_BP_Direct	GO: 0050853~B cell receptor signaling pathway	6	1.27	1.90E-04
GOterm_BP_Direct	GO: 0007169~transmembrane receptor protein tyrosine kinase signaling pathway	7	1.48	9.83E-04
GOterm_BP_Direct	GO: 0007229~integrin-mediated signaling pathway	7	1.48	1.16E-03
GOterm_BP_Direct	GO: 0032467~positive regulation of cytokinesis	5	1.06	1.79E-03
GOterm_BP_Direct	GO: 0007626~locomotory behavior	7	1.48	2.27E-03
KEGG_Pathway	ptr04062: Chemokine signaling pathway	12	2.53	2.18E-03
KEGG_Pathway	ptr04060: Cytokine-cytokine receptor interaction	13	2.75	4.61E-03
KEGG_Pathway	ptr04115: p53 signaling pathway	7	1.48	5.37E-03
KEGG_Pathway	Primary immunodeficiency	5	1.06	8.66E-03
KEGG_Pathway	ptr04640: Hematopoietic cell lineage	7	1.48	9.54E-03

Supplemental table 3. Drug screening docking analysis of medicine of SYK

ID	Lib Dock Score
ZINC000095099885	145.056
ZINC000008215434	141.143
ZINC000008216447	128.681
ZINC000096014967	127.631
ZINC43131420	124.859
ZINC000028538573	120.091
ZINC000196105913	119.772
ZINC000072131515	117.381
ZINC000014883350	116.204
ZINC000008219152	116.156
ZINC000008216371	113.707
ZINC000008551971	113.138
ZINC000008215403	113.126
ZINC000001540640	112.792
ZINC000008217042	111.322
ZINC000013130935	109.746
ZINC000002526388	109.297
ZINC000100774817	109.069
ZINC000014883348	109.031
ZINC000030729923	108.771

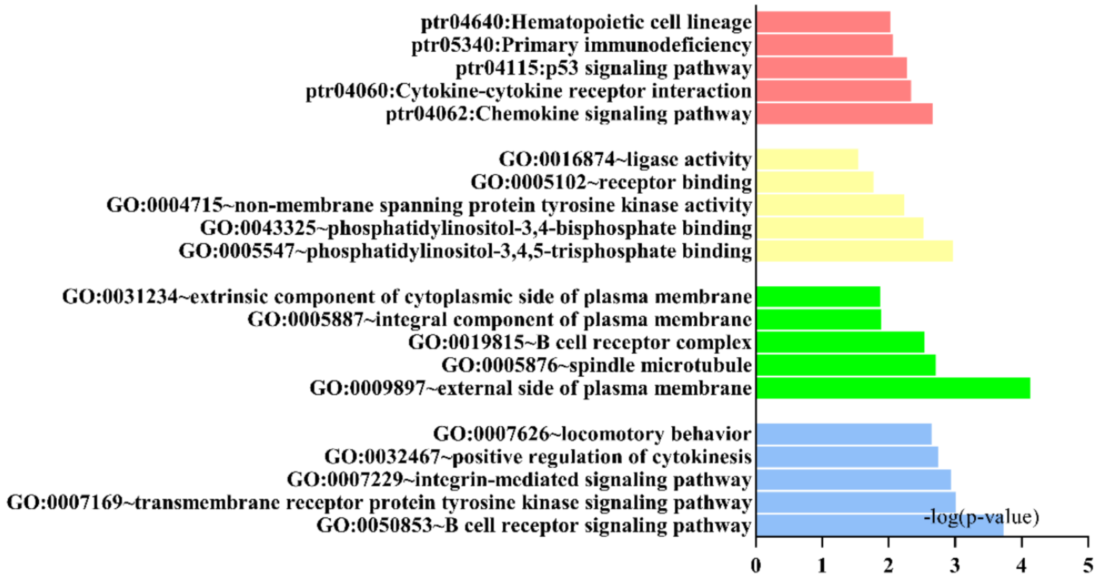

Supplemental Fig.1 Results of gene ontology (GO) and Kyoto Encyclopedia of genes and genome (KEGG).

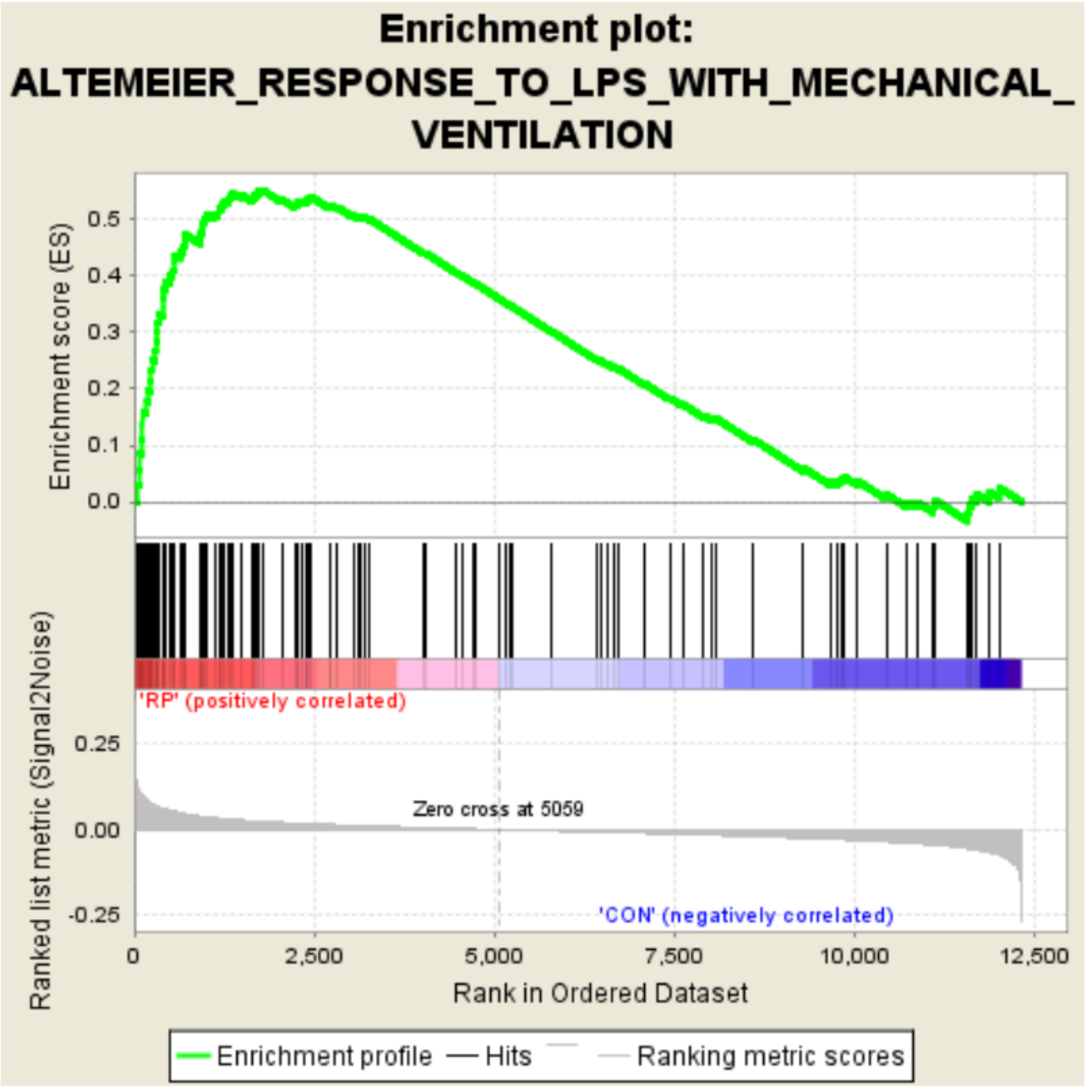

Supplemental Fig.2 Results of Gene Set Enrichment Analysis indicated abnormal regulation of mechanical ventilation pathways in radiation pneumonia

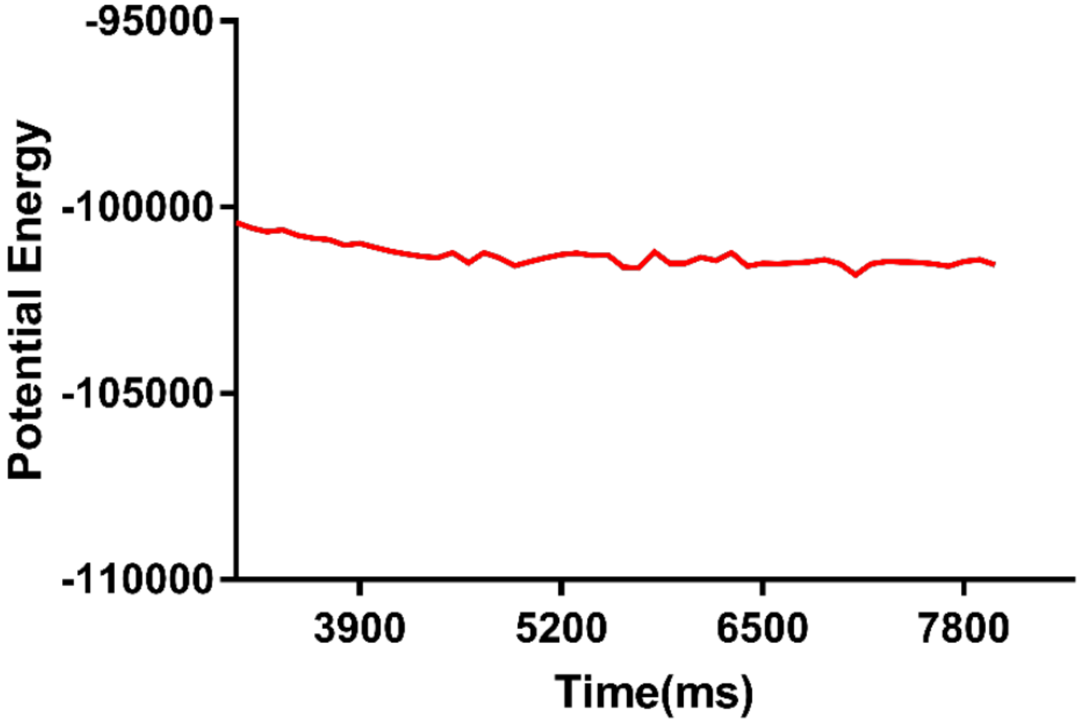

Supplemental Fig.3 Potential energy between ZINC43131420 and SYK transcriptional protein
